# Supplementary material for: Mechanisms governing the pioneering and redistribution capabilities of the non-classical pioneer PU.1
Source: Nat Commun. 2020 Jan 21;11:402. doi: 10.1038/s41467-019-13960-2 (PMC6972792; doi:10.1038/s41467-019-13960-2)
Supplement: Supplementary file 7 — Source data [file 41467_2019_13960_MOESM7_ESM.zip › Source_Data/Figure6/Figure6B_MotifScanOutput/FLI1_mutPU.1/homerResults/motif15.similar.html]

motif15

## Information for motif15

G
T
A
C
C
G
T
A
C
T
A
G
A
G
T
C
C
G
A
T
A
C
T
G
G
C
T
A
G
A
T
C
C
T
G
A
  
Reverse Opposite:  

G
A
C
T
C
T
A
G
C
G
A
T
A
G
T
C
G
C
T
A
A
C
T
G
A
G
T
C
C
G
A
T
C
A
T
G
  

|  |  |
| --- | --- |
| p-value: | 1e-87 |
| log p-value: | -2.008e+02 |
| Information Content per bp: | 1.637 |
| Number of Target Sequences with motif | 4419.0 |
| Percentage of Target Sequences with motif | 43.07% |
| Number of Background Sequences with motif | 12365.5 |
| Percentage of Background Sequences with motif | 32.44% |
| Average Position of motif in Targets | 100.6 +/- 56.5bp |
| Average Position of motif in Background | 101.1 +/- 64.8bp |
| Strand Bias (log2 ratio + to - strand density) | 0.0 |
| Multiplicity (# of sites on avg that occur together) | 1.22 |
| Motif File: | file (matrix) reverse opposite |

### Similar de novo motifs found

|  |  |  |  |  |  |  |  |
| --- | --- | --- | --- | --- | --- | --- | --- |
| Rank | Match Score | Redundant Motif | P-value | log P-value | % of Targets | % of Background | Motif file |
| 1 | 0.767 | G C A T A T C G T C G A A G T C C G T A C A T G G A T C A C G T C A T G G C T A | 1e-60 | -139.175573 | 17.26% | 11.02% | motif file (matrix) |
| 2 | 0.754 | A G T C C G A T A T G C C G T A C T A G A G T C G T C A A C T G | 1e-36 | -83.444031 | 15.71% | 11.00% | motif file (matrix) |
